# Prevalence rates of childhood trauma in medical students: a systematic review

**DOI:** 10.1186/s12909-017-0992-2

**Published:** 2017-09-12

**Authors:** Eimear King, Claire Steenson, Ciaran Shannon, Ciaran Mulholland

**Affiliations:** 1 Centre for Medical Education, Department of Psychiatry, Queen’s University of Belfast, 97 Lisburn Road, Belfast, Northern Ireland BT9 7BL UK; 2 School of Psychology, Queen’s University of Belfast, Belfast, Northern Ireland UK

**Keywords:** Medical students, Mental health, Childhood trauma

## Abstract

**Background:**

It is known that medical students suffer from high rates of mental health difficulties. In recent years there has been an increasing focus on the need to improve support and treatment services for those in difficulty. In order to meet these needs it is important to clarify the relevant aetiological factors. There is robust evidence from general population studies that a history of childhood trauma (including physical and sexual abuse and emotional neglect) predisposes to the subsequent development of mental health difficulties in adult life. It has previously been speculated that students with a history of such trauma might preferentially apply to study medicine.

**Methods:**

This systematic review seeks to examine the existing evidence base with regard to rates of childhood trauma in medical student populations. Articles were identified through a literature search of psychINFO, web of science, Embase and medline.

**Results:**

This search generated 11 articles which were deemed to meet criteria for inclusion in this review. There is a wide range of results given for rates of childhood trauma in these studies.

**Conclusions:**

The published research which examines rates of childhood trauma affecting medical students is limited and difficult to generalise from, or to use to draw firm conclusions. Given the possible negative outcomes of a history of childhood trauma in medical students, including that such a history may be associated with difficulties in a student progressing in their undergraduate and postgraduate examinations, well-organised prospective studies are required.

## Background

Most of the published literature suggests that medical students suffer higher rates of mental health difficulties than other students, and the general population. For example, when Dyrbye and colleagues carried out a systematic review of 40 American and Canadian studies published between 1980 and 2005 they found clear evidence of a higher prevalence of depression and anxiety among medical students than in the general population [1]. Whilst some recent UK studies did not find that mental health difficulties are more common in medical students-a study in Cambridge found prevalence rates of depression between 2.2 and 14.8%, matching that of the general population [2], a study at University College London, found that non-medical students had higher rates of moderate to severe depression than medical students [3] and a study at Edinburgh, found similar rates of psychological morbidity in medical and non-medical students [4]-these are at variance with the main body of literature.

One result of increasing awareness of this evidence base has been a sharper focus on the mental health needs of medical students. In the UK the General Medical Council (GMC) document *Supporting Medical Students with Mental Health Conditions* highlights the considerable stress suffered by medical students and the need for improved support services [5]. In order to provide such services and relevant and effective therapeutic interventions it is essential to establish the important aetiological factors which predispose to mental health problems in medical students. One such well-established aetiological factor in general population studies is a history of childhood trauma (CHT) with a large body of evidence supporting a link with the subsequent development of mental health difficulties in adult life [6]. It has previously been speculated that students with a history of such trauma might preferentially apply to study medicine as they may view the prospect of caring for others as therapeutic for themselves, either consciously or sub-consciously [7]. There are possible negative outcomes of a history of CHT in medical students: a history of CHT may be associated with difficulties in a student progressing in their undergraduate and postgraduate examinations [8], for example. This systematic review seeks to examine the existing evidence base with regard to rates of CHT in medical student populations.

## Methods

### Search strategy and selection criteria

Articles were identified through a literature search of psychINFO, web of science, Embase and medline using the following search terms: child abuse, child sexual abuse, child neglect, physical abuse, emotional abuse, domestic violence, adult survivors of child abuse, post-traumatic stress disorder, medical student, undergraduate medical education. Searches were adapted for the different databases with Boolean operators ‘AND’ and ‘OR’. The search was implemented separately by two reviewers using the procedure outlined by Higgins and Green [9].

Additional references were retrieved by cross-referencing of selected articled. Disagreement was resolved through discussion between the investigators.

Studies were included in this review if they met the following inclusion criteria:

(1) Original research only.

(2) Studies from any date of publication.

(3) Full text in English only.

(4) Participants to include medical students.

(5) Quantitative studies with a prevalence rate of CHT specifically for medical students.

(6) The trauma to have occurred before the age of 18.

### Study selection

A total of 735 articles were returned from the initial search. Two investigators (EK and CSt) independently reviewed all the articles with any disagreement being resolved through discussion involving all authors. The strength of agreement between the two raters was high (kappa = 0.78).

Of the 735 articles, 385 were removed as they were duplicates. The remaining 350 articles were screened against the inclusion and exclusion criteria. A total of 121 articles were excluded at title, a further 137 excluded at abstract and 81 excluded after the full text was read.

Reasons for exclusion after examining full texts included: papers presenting results which included trauma not specifically occurring before the age of 18; papers from which it was not possible to determine a prevalence rate related exclusively to medical students; studies which were qualitative in nature and therefore did not provide a prevalence rate.

This search generated 11 articles which were deemed to meet criteria for inclusion in the study. The references lists of these articles were examined but this do not lead to any further articles being included.

### Quality assessment

A quality assessment checklist was applied to each of the studies, based on the proforma created by Kmet, Lee and Cook [10]. Criteria 5–7, applicable to studies assessing interventions, were removed. Articles were assessed for quality independently by two reviewers in an effort to improve reliability. Results of the quality assessment are presented in Table 1.

**Table 1:** Summary of studies included in review

Author(s) and year of publication	Country of study	Year(s) of data collection	Number of students	Type of CHT	Use of validated assessment tool	Quality score (/22
Orhon FS et al. (2006) [11]	Turkey	2002	106	Physical	No	19
Xiao Q et al. (2008) [12]	China	2004–2005	2073	10 categories including emotional, physical, sexual and parental discord	Incorporated questions from the Conflict Tactics Scale (CTS) and the Childhood Trauma Questionnaire (CTQ)	20
Delahunta ET, Tulsky AA (1996) [13]	USA	1995	217	Physical and sexual	No	16
Ambuel et al. (2003) [18]	Mid west USA	Unclear from paper	223	Physical and sexual	Incorporated questions from the CTS	20
Cullinane PM, Alpert EJ, Freund KM (1997) [15]	USA	1991–1992	370	Physical and sexual	Incorporated questions from the CTS	21
Usta et al. (2014) [20]	Lebanon	2009	545	Physical and verbal	CTS	20
Kumaraswamy et al. (2010) [19]	Malaysia		196	Physical	Discipline questionnaire	16
Aydin S (2006) [21]	Kazakhstan/turkey	2003	287	Physical and sexual	No	9
Jeon HJ et al. (2009) [16]	Korea	2007	6986	Physical, emotional, Sexual, general trauma	Early trauma inventory self-report – short form	22
Haj-Yahia MM, Zoysa PD (2008) [17]	Sri Lanka	2007	476	Physical	CTS	17
Burazeri et al. [14]	Albania	2010	2797	Witnessed violence	Adapted from the reproductive health questionnaire	18

*CTS* conflict tactics scale, *CTQ* childhood trauma questionnaire

## Results

Table 1 provides a summary of the 11 articles including: the geographical area where the study took place; the number of participants in the study; whether an objective validated assessment tool was used; and the rate of CHT established by the study [11–21]. The quality of each study is included in the final column. The number of study participants ranged from 106 to 6986 with a median number of 370. Five studies were carried in the last 10 years.

A narrative review is presented as marked differences between studies made the combination of results inappropriate. The narrative analysis includes comparison of data where possible and an appraisal of study quality. For the purposes of this paper, we have organised the studies into those seeking to determine whether or not there was a history of physical abuse, sexual abuse and other forms of abuse. Studies could of course belong to more than one, or all categories.

### Rates of childhood physical abuse in medical students

Ten out of the 11 studies investigated rates of childhood physical abuse (Table 2). The study which did not generate a rate of physical abuse measured ‘witnessed violence by the father directed towards the mother’ [11].

**Table 2:** Rates of childhood physical abuse

Study (Reference)	Rate of physical abuse (%)	Quality score
Orhon FS et al. (2006) [11]	65	19
Xiao Q et al. (2008) [12]	26.7	20
Delahunta ET, Tulsky AA (1996) [13]	12.9	16
Cullinane PM, Alpert EJ, Freund KM (1997) [15]	7	21
Jeon HJ et al. (2009) [16]	9.4	22
Haj-Yahia MM, Zoysa PD (2008) [17]	12	17
Ambuel et al. (2003) [18]	30	20
Kumaraswamy et al. (2010) [19]	5	16
Aydin S (2006) [21]	26.5	9
Usta et al. (2014) [20]	22	20

The rates reported ranged from 5 to 65%. Two of the studies did not use a validated questionnaires but rather asked the participant to answer ‘yes’ or ‘no’ to the question ‘I was physically abused as a child’ [12, 13].

### Rates of childhood sexual abuse in medical students

Six of the 11 studies provide rates of sexual abuse (Table 3). One study [12] asked participants to respond to the question ‘as a child did you experience sexual abuse?’ without defining sexual abuse. Once this study is excluded the rates of CSA reported range from 2.9% to 13% across the other 5 studies. One study [13] produced two different rates of sexual abuse (6% and 13%) for abuse perpetrated by a family member and abuse perpetrated by a non-family member respectively. The other five studies did not make any distinctions regarding who carried out the abuse.

**Table 3:** Rates of childhood sexual abuse

Study	Rate (%)
Xiao Q et al. (2008) [11]	8.3
Delahunta ET, Tulsky AA (1996) [12]	9.7
Cullinane PM, Alpert EJ, Freund KM (1997) [14]	9
Jeon HJ et al. (2009) [18]	2.9
Ambuel et al. (2003) (13	13
Aydin S (2006) [17]	35.9

### Rates of other trauma in childhood in medical students

Two of the studies [12, 16] looked at types of CHT other than physical, sexual and emotional abuse. One measured 10 different types of CHT [12] including experience of mental illness in the household, emotional neglect, parental discord, and experience of a family member being put in jail. This study generated a high quality score based on the large number of participants and the use of a validated questionnaire. Rates of physical neglect and physical abuse were significantly higher than emotional abuse (3.9%). The rate of substance abuse was 15.3% [16]. The rate of reported parental discord was 5.4%. It is reasonable to assume that there was an overlap between the experiences of different forms of abuse. The second study [14] recorded rates of ‘general trauma’ (this included eight different types) and gave an overall rate of 13.2%.

## Discussion

The existing research examining rates of CHT in medical student populations is limited both in quantity and quality. It is thus difficult to synthesis the findings or to generalise from them. Though we adopted a robust approach to quality the weakness of the evidence base meant that this did not help to clarify the situation to a significant extent. Studies of higher quality were those which: provided sufficient baseline demographic information relating to the participants; used validated assessment tools; had clearly defined thresholds for the presence of a history of CHT; and had larger, more representative samples. There are however insufficient high quality studies at this time.

A wide range of results for rates of CHT is reported in the studies but this is not surprising for a number of reasons. Study quality and methodologies varied considerably, for example, even something as important as what is considered to constitute CHT was not defined in ways that allowed clear comparisons between studies (and two studies did not define abuse in any way [12, 13]). As all the studies were retrospective in design there is an obvious potential problem with recall bias. There are also issues around subjectivity though efforts have been made in some of the studies to use objective and validated assessment tools with clear definitions of abuse (including the Childhood Trauma Questionnaire, Traumatic Events Survey, and the Early Trauma Inventory Self Report). Even with the use of such tools what one individual categorises as traumatic, others may not, and indeed what an individual considers to be an abusive childhood experience may reflect cultural variation in the perception of what constitutes such an experience [22].

A further problem is that it is possible that medical students are more reluctant to disclose abusive experiences than most people, even when anonymity is guaranteed, despite the recent societal shift in attitudes towards the reporting of CHT. A particular reluctance to disclose CHT among the medical student population may arise because of a perception that such disclosure may in some way impede progress in their medical career. The possibility of an increasing willingness to disclose abusive experiences in childhood across wider society is demonstrated by the similar studies carried out in 1999 and 2009 by the NSPCC: the 1999 study examined the rate of ‘childhood maltreatment’ in the UK (in a randomly selected group of 2869 people aged between 18 and 24 years) found a rate of 16% [23] whereas the second study (2009) found that 25.3% (of a randomly selected group of 1761 people aged between 18 and 24 years) described childhood maltreatment [24]. A meta-analysis carried out in 2008 looked at the prevalence of childhood sexual abuse (CSA) around the world in student and community samples (included 65 studies from 22 countries) and showed that 7.9% of men and 19.7% of women had suffered some form of CSA [25]. It is likely that medical students will become more willing to disclose CHT in line with changes in societal attitudes.

Students who receive the appropriate support at medical school are more likely to do well. An improved understanding of the likelihood of students having experienced CHT, and of its potential consequences on well-being and educational outcomes, is essential for educators engaged in pastoral care. The demands of the medical undergraduate course are significant and students with a history of CHT may be more vulnerable to academic stress [26]. Significant rates of alcohol and drug use exist among medical students [27] and there is evidence suggesting a link between drug dependency and a history of CHT [28].

A history of CHT is not only associated with negative outcomes however. Students who have experienced their own traumas may find it easier to broach more difficult topics in the consultation. It is interesting that in a study in Massachusetts medical professionals who had experienced abuse were more confident in screening for abuse amongst those they treated [29].

## Conclusions

The current research base examining rates of childhood trauma in medical student populations is very limited. Given the possible negative outcomes of a history of childhood trauma in medical students, including that such a history may be associated with difficulties in a student progressing in their undergraduate and postgraduate examinations, well-organised, large-scale prospective studies are required to clarify both the rates of such trauma, and the potential impact on future well-being and academic progression.

### Strengths and limitations

CHT is a sensitive topic. Participants in research studies may not be ready to disclose traumas, or choose not to. This may be a particular problem for medical students who are often concerned about the implications of showing signs of “weakness” or to “admitting” to mental health problems.

The systematic review excluded studies in any language other than English. This may mean some relevant articles in a different language were excluded.

The studies to date have been completed over a wide timescale, during which attitudes to the reporting of CHT have changed, and have been carried out in very different societal contexts.
